# A Quasi-Experimental Study on the Effectiveness of Compulsory and Voluntary Treatment Settings for 1,299 Drug Abusers in Hunan, China

**DOI:** 10.3389/fpsyt.2021.613665

**Published:** 2021-08-27

**Authors:** Kai Huang, Caihua Yu, Xinxin Chen, Yuzhu Hao, Yudan Ding, Zhenzhen Wu, Xuyi Wang

**Affiliations:** ^1^Second Xiangya Hospital, Central South University, Changsha, China; ^2^Division of Research on Stress and Addiction Research, Second Xiangya Hospital, Central South University, Changsha, China; ^3^Department of Psychiatry, Second Xiangya Hospital, Central South University, Changsha, China; ^4^Key Laboratory of Psychiatry and Mental Health of Hunan Province, Central South University, Changsha, China; ^5^Mental Health Institute, Second Xiangya Hospital, Central South University, Changsha, China; ^6^National Clinical Research Center for Mental Disorders, and Department of Psychaitry, The Second Xiangya Hospital of Central South University, Changsha, China

**Keywords:** drug addicts, compulsory drug rehabilitation centers, voluntary drug rehabilitation centers, relapse, family support

## Abstract

**Background:** Although the type and structure of substance abuse treatment have changed, the overall approaches of drug rehabilitation in China has remained largely unchanged. Evidence of effectiveness for compulsory drug rehabilitation centers (CRCs) and voluntary drug rehabilitation centers (VRCs) remains mixed. The main objective of our study is to reveal the outcomes of CRCs and VRCs and examine the factors associated with relapse in these two centers.

**Methods:** In this cross-sectional study, we recruited a total of 1,299 drug abusers in Hunan Province, 709 from CRCs and 590 from VRC, respectively. We used Pearson chi-squared test and *t*-test to examine the differences in demographics and drug-related characteristics. Binary logic regression was used to examine the relationship between important factors and relapse in CRCs and VRC.

**Results:** Patients from CRCs and VRC significantly differed in age, sex, types of drug used, medical illness, education, occupation, mental illness, and marital status. After drug rehabilitation, both groups both had improved in occupation, family support, and social function (*p* < 0.05). In addition, employment and family support were significantly associated with a decreased risk of relapse (*p* < 0.05).

**Conclusion:** This study revealed that compulsory rehabilitation is as effective as voluntary rehabilitation in (1) getting jobs and increasing monthly income, (2) having a good relationship with family, and (3) becoming more satisfied with their spared time. The components of these two settings were very different and may imply the necessity of these two approaches in China. In addition, employment and family support may prevent relapse.

## Introduction

Drug abuse has been a serious problem in China since the 1980s. According to the China National Narcotic Control Commission (CNNCC) report, the current number of drug users in China accounts for 0.18% of the country's total population. At the end of 2018, there were 2.404 million drug addicts nationwide (1). Although China has achieved some positive results in controlling drug abuse, the abuse of synthetic drugs is still spreading. To effectively decrease the number of drug addicts, China has been promulgating the Drug Control Law of the People's Republic of China since December 29, 2007. China's latest Drug Control Law suggests that first-time drug addicts can choose to recover in their local residential communities. On the other hand, relapsed drug addicts have to receive rehabilitation in isolated compulsory detoxification centers (ICDCs) for 2 years (possibly 1–3 years, based on the degree of recovery). Released drug addicts from the ICDCs may recover in their local residential communities (2).

To date, there are four main drug abuse treatment methods in China: compulsory drug rehabilitation centers (CRCs), voluntary drug rehabilitation centers (VRCs), community detoxification, and community rehabilitation. Compulsory drug rehabilitation forcibly isolates qualified drug abusers to a rehabilitation facility after due legal procedures. Voluntary drug rehabilitation refers to the voluntary choice of community drug rehabilitation or qualified medical institutions for drug rehabilitation. Community detoxification places qualified drug addicts in the community. The primary level organization for community detoxification includes community workers, public security, and the public to help drug abusers eliminate drug addiction and restore typical family and social functions. For community rehabilitation, the government in conjunction with medical institutions and social forces sets up rehabilitation centers in the community and refers drug addicts to them to prevent relapse after they leave other rehabilitations or detoxifications.

While CRCs and VRCs account for the vast majority of China's rehabilitation centers, controversies surround efficacy in both approaches. CRCs have been criticized for various potential human rights abuses, including restricting personal freedom and forcing addicts to do manual labor. Besides, some researchers do not believe that it may decrease relapse for drug addicts compared to other methods (3). A study in Vietnam found that in addition to being less effective than voluntary drug rehabilitation in achieving drug-free days, compulsory drug rehabilitation is also more expensive (4). In a later study (5), compulsory drug rehabilitation was found to be less effective than voluntary drug rehabilitation in reducing heroin use, monthly drug expenditure, blood-borne virus risky behaviors, and drug-related illegal behaviors. Norwegian scholar Pasareanu et al. believe that although compulsory and voluntary rehabilitation may reduce the mental distress, patients in compulsory rehabilitation have a great risk of rebound and need post-rehabilitation interventions to prevent relapses and mental distress (6). As early as 2012, 12 United Nations agencies, including the World Health Organization, issued a joint statement calling on Member States to close compulsory drug detention and rehabilitation centers and recommended voluntary, informed, and rights-based health and social services in communities, while one of the scholars from the Chinese Center for Disease Control and Prevention expressed support for the argument of compulsory treatment for opioid dependence. He believes that mandatory treatment for opioid dependence should be an integral part of a broader harm reduction strategy that includes voluntary treatment, needle exchange programs, voluntary counseling and testing, expanded infectious disease treatment coverage, peer outreach, and intensive educational campaigns. The role of mandatory treatment centers is to protect opioid addicts and their communities and to provide an essential means of assisting opioid addicts who repeatedly refuse outpatient treatment and engage in criminal activities (7). Others also believed that compulsory treatment centers might reduce relapse rates in China (8).

In China, drug addicts in CRCs receive detoxification treatment, physical medical care, behavioral therapy, moral and legal education, drug and health education, skills training, discipline training, physical exercise, and manual labor (9, 10). Group treatment has been adopted in China by integrating various individual and group treatment techniques for drug addicts in CRCs (11, 12). Moreover, virtual reality aversion therapy was applied in CRCs to reduce drug craving (13). At present, CRCs in China have weakened the punishment and highlighted their therapeutic functions. Different treatment measures were considered based on the sex, age, and other conditions of drug addicts. Users of different drugs were also separated to avoid cross-infection. Drug addicts are also treated in different stages according to their degree of addiction. Most importantly, all of treatment in CRCs is free.

In summary, evidence regarding the effectiveness of CRCs and VRCs remains inconsistent. Therefore, more research is needed to draw solid conclusions about the effectiveness of drug abuse treatment in CRCs and VRCs. Our study was conducted to assess the effectiveness of CRCs and VRCs and explore the predictors of relapse in CRCs and VRCs in China.

## Methods

### Study Design and Procedures

A quasi-experimental study design was used to test the effectiveness of two treatment settings for drug addicts: compulsory isolation drug rehabilitation centers and voluntary drug rehabilitation centers in Changsha, China. The Ethics Committee of the Second Xiangya Hospital of Central South University approved the research. No monetary compensation or other incentives were provided to the participants. The procedures are as follows: (1) collect all clinical records, demographic and social characteristics, lifetime and recent drug use, social support, and drug-related psychosis of patients released from two compulsory centers and one voluntary center; (2) follow-up telephone interview about family relationships, physical disorders, and relapse situations; and (3) exclude participants who did not complete the interview or provide complete information.

### Participants

Participants recruited in this study were drug addicts released between December 1, 2016, and December 30, 2018, from two treatment settings in Changsha, a city in China. Some came from CRCs: Hunan Baimalong and Xinkaipu CRCs, the two major treatment centers for detoxifying arrested drug users. Others came from Hunan Kangda VRC. Both compulsory and voluntary settings are restrictive environments with different structural and treatment procedures used to control substance addiction.

**Figure 1:**
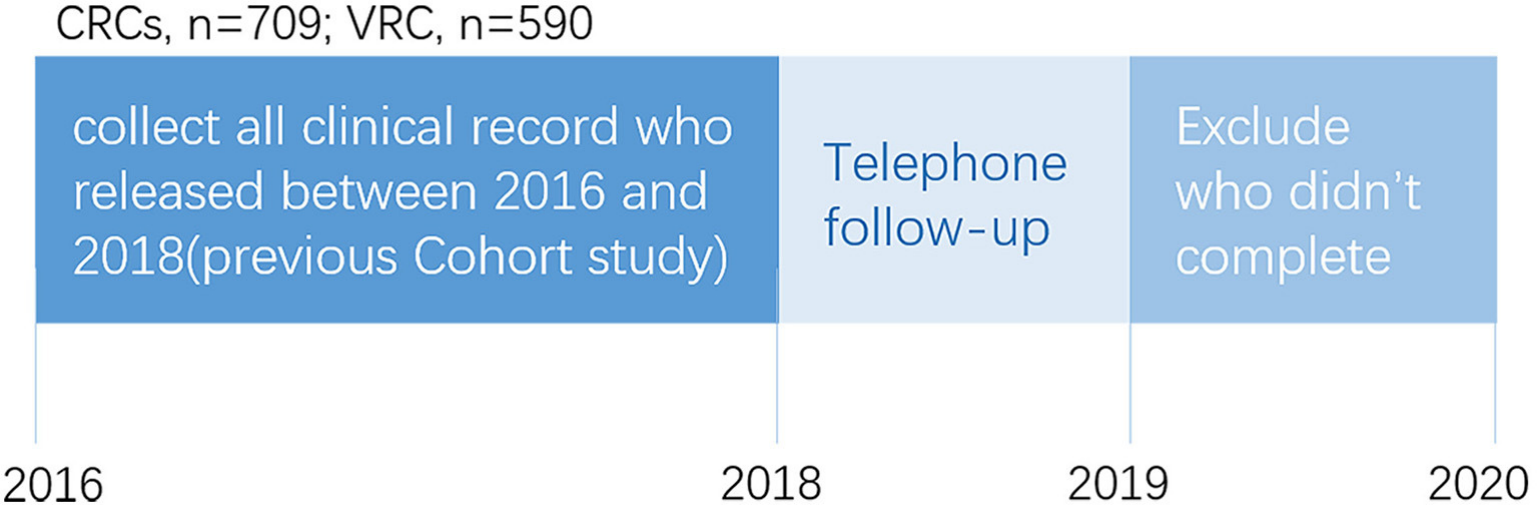
The overall profile of survey.

We randomly sampled 1,299 drug abusers from CRCs and VRCs: 709 from CRCs and 590 from VRCs. The criteria of this study are the following: (1) admitted drug abusers to the rehabilitation centers; (2) capable of effective communication; (3) over 16 years old; and (4) all mental diseases are meeting DSM-IV criteria. Participants were guaranteed that all personal information was strictly confidential. After the telephone follow-up, 776 drug abusers agreed to complete our questionnaires. Thus, the final sample of our study includes 337 from CRCs and 439 from VRCs (see Figure 1).

### Statistical Analyses

All statistical analyses were performed using the software SPSS version 25.0. Descriptive statistics were used to report group general information and participant variables. Pearson chi-squared test and *t*-test were used to examine the differences in demographics and drug use-related characteristics. Binary logic regression was used to examine the relationship between important factors and relapse in CRCs and VRCs. All tests were two-sided, with *p* < 0.05 to be considered statistically significant in this study.

## Results

### The Characteristics of Participants in CRCs and VRCs

After an initial screening, 776 drug addicts in Changsha, Hunan Province, China (439 in CRCs and 337 from VRCs) were included in this study. Of all the missing participants, 496 were untraceable, had incomplete information, or had absence of communication. The non-response rates in CRCs (52.5%) were twice the rates in VRCs (25.5%). The demographic data and drug-related characteristics of participants are shown in Table 1.

**Table 1:** The characteristics and mental health of participants in CRCs and VRCs (*N* = 776).

**Characteristic, *n* (%)**	**CRCs (*n* = 337)**	**VRCs (*n* = 439)**	**F/χ^**2**^**	***P*-value**
Age (mean ± SD years)	35.18 ± 7.92	30.89 ± 7.01	14.817	<0.001^***^
Sex			60.644	<0.001^***^
Male	250 (74.2%)	413 (94.1%)		
Female	87 (25.8%)	26 (5.9%)		
Drug type			96.575	<0.001^***^
Methamphetamine	246 (73.0%)	384 (87.5%)		
Opiate	67 (19.9%)	4 (0.9%)		
Both	14 (4.2%)	8 (1.8%)		
Others	10 (3.0%)	43 (9.8%)		
Drinking^▴^			52.894	<0.001^***^
Yes	197 (68.2%)	177 (40.6%)		
No	92 (31.8%)	259 (59.4%)		
Smoking^▴^			3.161	0.075
Yes	225 (83.3%)	384 (88.1%)		
No	45 (16.7%)	52 (11.9%)		
Physical illness^▴^			11.057	0.001^**^
Yes	63 (20.1%)	136 (31.0%)		
No	250 (79.9%)	303 (69.0%)		
Education			115.444	<0.001^***^
Primary school or below	82 (24.3%)	7 (1.6%)		
Junior middle school	186 (55.2%)	339 (77.2%)		
Senior high school	60 (17.8%)	54 (12.3%)		
College or above	9 (2.7%)	39 (8.9%)		
Occupation^▴^			28.322	<0.001^***^
Yes	95 (31.7%)	221 (51.5%)		
No	205 (68.3%)	208 (48.5%)		
Monthly income^▴^			8.188	0.042^*^
0-1,999 Yuan	215 (83.3%)	212 (80.9%)		
2,000-3,999 Yuan	26 (10.1%)	16 (6.1%)		
4,000-5,999 Yuan	7 (2.7%)	12 (4.6%)		
≥6,000 Yuan	10 (3.9%)	22 (8.4%)		
Marital status			34.075	<0.001^***^
Never married	133 (39.5%)	133 (30.3%)		
Married	129 (38.3%)	256 (58.3%)		
Divorced or widowed	75 (22.3%)	50 (11.4%)		
Mental health^▴^			8.336	0.004^**^
Yes	1 (0.3%)	15 (3.4%)		
No	308 (99.7%)	422 (96.6%)		

▴ *Some participants were excluded due to incomplete information*.

*Chi-squared test was performed for categorical, and t-test was performed for continuous variables*.

* *p < 0.05*,

** *p < 0.01*,

*** *p < 0.001*.

There were significant differences in demographics between participants from CRCs and VRCs. Participants from CRCs (35.18 ± 7.92) were older than those from VRCs (30.89 ± 7.01) (*F* = 14.817, *p* < 0.001). The majority of participants from both centers were male, especially in VRCs (94.1%) (χ^2^ = 60.644, *p* < 0.001), and the type of drug use were methamphetamine (CRCs: 73%; VRCs: 87.5%; χ^2^ = 96.575, *p* < 0.001). After excluding participants whose families and friends cannot provide correct information, significant differences were found in drinking problems (χ^2^ = 52.894, *p* < 0.001) and physical illness (χ^2^ = 52.894, *p* < 0.01). The educational level in both groups were predominantly junior middle school graduates (χ^2^ = 115.444, *p* < 0.001). Participants from VRCs were more likely to have employment than those from CRCs (χ^2^ = 53.286, *p* < 0.001). There was a significant difference in physical illness (χ^2^ = 11.057, *p* < 0.001). Participants from CRCs who were never married (17.1%) were similar to those who were married (16.6%), while most people were married (33.0%) in VRCs (χ^2^ = 34.075, *p* < 0.001). The vast majority of participants from both centers were not mentally ill (χ^2^ = 8.336, *p* < 0.01).

### The Outcomes of CRCs and VRCs

Relapse: We assumed that participants who did not follow-up with telephone interview were in relapse. The rates of relapse in participants from CRCs and VRCs were was 54.9 and 32.4%, respectively.

After treatment, participants from CRCs were more likely to find jobs after treatment (69.3 vs. 31.9%, χ^2^ = 88.054, *p* < 0.001). They were more likely to have a good relationship with family (63.2 vs. 25.8%, χ^2^ = 105.475, *p* < 0.001) and less conflict with family (4.7 vs. 26.7%, χ^2^ = 61.301, *p* < 0.001). Besides, they were more likely to have permanent homes (88.7 vs. 79.8%, χ^2^ = 9.818, *p* < 0.01) and more satisfied with their spare time (37.7 vs. 24.1%, χ^2^ = 18.474, *p* < 0.001). See Table 2 for more details.

**Table 2:** Comparison of related factors before and after rehab in CRCs.

**Characteristic, *n* (%)**	**Before treatment**	**After treatment**	**χ^**2**^**	***P*-value**
Occupation^▴^			88.054	<0.001^***^
Yes	96 (31.9%)	228 (69.3%)		
No	205 (68.1%)	101 (30.7%)		
Income monthly			60.454	<0.001^***^
0-1,999 Yuan	215 (83.3%)	117 (51.1%)		
2,000-3,999 Yuan	26 (10.1%)	62 (27.1%)		
4,000-5,999 Yuan	7 (2.7%)	32 (14.0%)		
≥6,000 Yuan	10 (3.9%)	18 (7.9%)		
Marital status			3.394	0.183
Never married	133 (39.5%)	114 (33.8%)		
Married	129 (38.3%)	130 (38.6%)		
Divorced or widowed	75 (22.3%)	93 (27.6%)		
Relationship with family			105.475	<0.001^***^
Good	87 (25.8%)	213 (63.2%)		
Normal	166 (49.3%)	103 (30.6%)		
Bad	84 (24.9%)	21 (6.2%)		
Conflict with family			61.301	<0.001^***^
Yes	90 (26.7%)	16 (4.7%)		
No	247 (73.3%)	321 (95.3%)		
Permanent home^▴^			9.818	0.002^**^
Yes	265 (79.8%)	297 (88.7%)		
No	67 (20.2%)	38 (11.3%)		
Living situation^▴^			2.238	0.327
Alone	53 (16.0%)	65 (19.4%)		
Family	253 (76.4%)	239 (71.3%)		
Others	25 (7.6%)	31 (9.3%)		
Do they have friends^▴^			0.025^*^	0.875
Yes	161 (50.6%)	164 (51.2%)		
No	157 (49.4%)	156 (48.8%)		
Satisfied with your spare time^▴^		18.474	<0.001^***^
Good	78 (24.1%)	114 (37.7%)		
Normal	159 (49.1%)	140 (46.4%)		
Bad	87 (26.9%)	48 (15.9%)		

▴ *Some participants were excluded due to incomplete information*.

*Chi-squared test was used in this table*.

* *p < 0.05*,

** *p < 0.01*,

*** *p < 0.001*.

For participants in the VRCs, participants were more likely to change from no employment to having a job (72.6 vs. 51.5%, χ^2^ = 40.977, *p* < 0.001) and bring home a higher monthly income home (χ^2^ = 55.092, *p* < 0.001) after rehabilitation. Relationship with family got better (51 vs. 12.5%, χ^2^ = 218.916, *p* < 0.001), conflict with family members was also reduced (10.7 vs. 55.1%, χ^2^ = 196.133, *p* < 0.001), and there was also an increase in satisfaction with their spare time (31.8 vs. 7.1%, χ^2^ = 98.322, *p* < 0.001). See Table 3 for more details.

**Table 3:** Comparison of related factors before and after rehab in VRCs.

**Characteristic, *n* (%)**	**Before treatment**	**After treatment**	**χ^**2**^**	***P*-value**
Occupation			40.977	<0.001^***^
Yes	221 (51.5%)	318 (72.6%)		
No	208 (48.5%)	120 (27.4%)		
Income monthly^▴^			55.092	<0.001^***^
0-1,999 Yuan	211 (80.8%)	126 (50.6%)		
2,000-3,999 Yuan	16 (6.1%)	35 (14.1%)		
4,000-5,999 Yuan	12 (4.6%)	48 (19.3%)		
≥6,000 Yuan	22 (8.4%)	4 0(16.1%)		
Marital status			3.660	0.160
Never married	133 (30.3%)	118 (26.9%)		
Married	256 (58.3%)	253 (57.6%)		
Divorced or widowed	50 (11.4%)	68 (15.5%)		
Relationship with family			218.916	<0.001^***^
Good	55 (12.5%)	224 (51.0%)		
Normal	210 (47.8%)	192 (43.7%)		
Bad	174 (39.6%)	23 (5.2%)		
Conflict with family^▴^			196.133	<0.001^***^
Yes	242 (55.1%)	47 (10.7%)		
No	197 (44.7%)	392 (89.3%)		
Permanent home^▴^			3.455	0.063
Yes	365 (92.6%)	418 (95.7%)		
No	29 (7.4%)	19 (4.3%)		
Live with^▴^			3.033	0.219
Alone	42 (10.7%)	32 (7.3%)		
Family	339 (86.3%)	389 (89.0%)		
Others	12 (3.1%)	16 (3.7%)		
Do they have friends^▴^			3.720	0.054
Yes	233 (59.3%)	281 (65.8%)		
No	160 (40.7%)	146 (34.2%)		
Satisfied with your spare time^▴^		98.322	<0.001^***^
Good	28 (7.1%)	139 (31.8%)		
Normal	275 (70.0%)	265 (60.6%)		
Bad	90 (22.9%)	33 (7.6%)		

▴ *Some participants were excluded due to incomplete information*.

^*^p < 0.05, ^**^p < 0.01,

*** *p < 0.001*.

### The Influence Factors of Relapse for Participants

Factors that influence relapse in both CRCs and VRCs were analyzed by backward binary logistic regression. Drug type, occupation, relationship with family, living with family members, and rehabilitation centers remain in the model as predictors of relapse rate. The odds of relapse for using other drug relapse rates were 4.5 times higher than using methamphetamine or/and opiates (OR: 4.583, 95% CI: 1.73–12.143). The odds of relapse rates were nearly three times higher for having no jobs after release than having jobs (OR: 2.702, 95% CI: 1.350–5.407). The odds of relapse for those who have normal (OR: 2.300, 95% CI: 1.117–4.738) and bad (OR: 6.523, 95% CI: 2.268–18.762) relationships with family after release were much higher than those who have good relationships with their families. Those who live with their families (OR: 0.312, 95% CI: 0.128–0.758) had lower odds of relapse than those who live alone or with others (Table 4).

**Table 4:** Binary logistic analysis for all participants from both CRCs and VRCs (*n* = 776).

**Factors**	**OR (95%CI)**	***P*-value**
Drug type		
Methamphetamine	1	
Opiate	1.13 (0.32-3.94)	0.854
Both	4.90 (0.85-28.11)	0.074
Others	4.58 (1.73-12.14)	0.002^**^
Occupation after release		
Yes	1	
No	2.70 (1.35-5.41)	0.005^**^
Relationship with family after release		
Good	1	
Normal	2.30 (1.12-4.74)	0.024^*^
Bad	6.52 (2.27-18.76)	0.001^**^
Living situation after release		
Alone	1	
Family	0.31 (0.13-0.76)	0.010^*^
Others	1.83 (0.46-7.33)	0.392
Rehabilitation centers		
CRCs	1	
VRCs	1.93 (0.87-4.24)	0.104
Constant	0.06	0.000

*Binary Logistic regression analysis was used to test the relationship with relapse. CRCs; VRC*.

* *p < 0.05*,

** *p < 0.01*.

For participants from CRCs, factors that influence relapse were analyzed by binary logistic regression. We directly carry out the conditional backward method of binary logistic regression with independent variables and dependent variables. The results are shown in Table 5. After regression, nine variables were excluded, including marital status, permanent home, have friends, and satisfaction with their spare time after release. There was a positive correlation between the working status, relationship with family, and conflict with family members after release. Those who have no jobs after release have higher odds of relapse (OR = 3.704). Normal (OR = 3.479) or bad (OR = 16.203) relationship with family and conflict with family (OR = 12.964) are also related to higher relapse.

**Table 5:** Binary Logistic regression analysis of factors to CRCs (*n* = 439).

**Factors**	**OR (95%CI)**	***P*-value**
Drug type
Methamphetamine	1	
Opiate	2.45 (0.92-6.52)	0.072
Both	5.45 (0.95-31.11)	0.057
Others	0	0.999
Occupation after release
Yes	1	
No	3.70 (1.45-9.46)	0.006^**^
Relationship with family after release
Good	1	
Normal	3.48 (1.32-9.14)	0.011^*^
Bad	16.20 (3.06-85.79)	0.001^**^
Conflict with family after release
No	1	
Yes	12.96 (1.17-144.24)	0.037^*^
Living situation after release
Alone	1	
Family	0.69 (0.24-1.93)	0.476
Others	3.61 (0.78-16.66)	0.100
Constant	0.24	<0.001^***^

*Binary Logistic regression analysis was used to test the relationship with relapse*.

* *p < 0.05*,

** *p < 0.01*,

*** *p < 0.001*.

Factors that influence relapse for the participants from VRCs were analyzed by binary logistic regression. The binary logical analysis model avoids multicollinearity by adopting the backward selection method and is suitable for determining the independent factors related to relapse in VRCs (Table 6). In the fitted model, drug types, occupation, marital status, permanent home, relationship with family members, conflicting with family, having friends, living with family, and satisfied with spare time after release were defined as initial covariates. After six step regressions, the final model found having no jobs (OR = 3.118), normal (OR = 3.126) or even bad (OR = 13.233) relationship with family, and other drug uses as clinical correlates of relapse. Furthermore, participants who lived with their family showed a negative correlation with relapse (OR = 0.339).

**Table 6:** Binary Logistic regression analysis of factors to VRCs (*n* = 439).

**Factors**	**OR**	***P*-value**
Drug type
Methamphetamine	1	
Opiate	1.41 (0.08-23.46)	0.812
Both	3.84 (0.59-24.75)	0.158
Others	4.34 (1.78-10.58)	0.001^**^
Occupation after release
Yes	1.00	
No	3.12 (1.64-5.94)	0.001^**^
Relationship with family after release
Good		
Normal	3.13 (1.50-6.52)	0.002^**^
Bad	13.23 (4.29-40.80)	<0.001^***^
Living situation after release
Alone	1.00	
Family	0.19 (0.07-0.47)	<0.001^***^
Others	0.34 (0.06-2.04)	0.238
Constant		<0.001^***^

*Binary Logistic regression analysis was used to test the relationship with relapse*.

^*^p < 0.05,

** *p < 0.01*,

*** *p < 0.001*.

## Discussion

This cross-sectional study used a self-compiled telephone follow-up interview to investigate and track general conditions and drug use, physical health, education and occupational functions, family and social support, and mental health of drug addicts. Participants from CRCs and VRCs were significantly differed in age, sex, types of drug used, medical illness, education, occupation, mental illness, and marital status. Compared with participants from VCR, participants from CRCs were older and had lower education level and income; participants in CRCs used more heroin and alcohol, and their marital status and mental illness were worse. The results suggested that CRCs and VRCs in China can provide appropriate services for the needs of different groups of drug abusers by focusing on different groups of drug abusers. Drug abusers with lower socioeconomic statuses and higher alcohol consumption were inclined to receive treatment from CRCs. In contrast, a relatively high number of individuals in VRCs suffered from physical diseases as well as mental illness. This may be related to China's compulsory detoxification policy. China's detoxification regulations are clear that drug users who suffer from acute infectious diseases or other serious diseases are not suitable for mandatory detoxification.

CRCs have been criticized for the potential for human rights abuses. Moreover, findings on the effectiveness of CRCs remained mixed. A study from Malaysia suggested that heroin-dependent drug addicts in compulsory centers are more likely to relapse after release than those treated in voluntary centers (5, 14). Previous studies in China have also shown a high risk of relapse in CRCs (15, 16). In addition, many also reported sexually transmitted diseases (STDs) in CRCs in some countries (17, 18). The Bangkok survey found that only 49.5% of drug addicts who had been quarantined in CRCs had not been injected again after being released for a year (19).

Though the rates of relapse in participants from CRCs and VRCs were different (54.9 and 32.4%), in this study, reductions in substance use, improvements in occupation, relationship with family, and satisfaction with their spare time were observed at both centers. In other words, CRCs are as effective as VRCs in reducing substance use and improving social integration. This was also found in previous research studies (4, 5, 20). Why did CRCs in China seem more efficacious? Compared to other countries, there are some advantages of compulsory drug rehabilitation in China: (1) The mode of compulsory drug rehabilitation in China has long been different from the previous means, and many changes have occurred. Some include specific psychological treatment (21), “three phase four zones” for improving social functions (22), etc., which have helped improve drug rehabilitation. (2) Compulsory drug rehabilitation is free of charge. (3) Current treatment methods are diverse, including physical therapy, psychological therapy, and employment support. (4) Different treatment methods were adopted for different ages, genders, and drug types; treatment was also graded and staged. Drug addicts from CRCs in China were influenced through ideological education, psychological or medical treatments, military training, and social production to recognize their deviant behaviors and prevent relapse (23). In some regions, the Labor and Employment Security Bureau selects professional members to train drug addicts for employment. There is a strict evaluation system for drug addicts to improve their abilities and adapt better to society. Some rehabilitation centers even increase opportunities for cooperation with social enterprises. Our results confirmed that employment rates were higher after treatment in CRCs.

Participants from both CRCs and VRCs reported lower relapse rates if they had stable jobs, a good relationship with family, and lived with them after release. Drug addicts who do not have work or are accompanied by bad family relationships may go back to find drug addict friends and then relapse. Those who have good family support can feel their warmth and care, and therefore have a higher sense of responsibility for themselves and their families, reducing the probability of relapse. Previous studies have shown that social support may help reduce relapse (24). For example, interventions that promote family cohesion are protective factors against relapse (25). Family support help drug addicts on their journey to recovery (26). Similarly, a previous study showed that family treatment could improve care for drug addicts and prevent relapse (27). Individuals who have volunteered or have paid employment are considered successful in recovery (28). In contrast, unemployment may cause drug addicts to question their self-worth and value (29).

## Limitation

Despite the insight into CRCs and VRCs, this study has several limitations. First, the samples were collected mainly in one city and one province in China and may not represent the national situation in China. Second, this was a cross-sectional study that does not allow the determination of cause-and-effect associations. Third, the participants' demographic characteristics from CRCs and VRCs were different; hence, we cannot compare the data between the two groups directly and assess which center had better outcomes. Fourth, in this study, the judgment of relapse was based on self-report and informant reports (family members) without a urine test. In order to ensure the authenticity, the report from family members shall prevail. Finally, there were high rates of non-response of participants from both CRCs and VRCs. This was not unusual in a follow-up study of drug abusers. We assumed that participants who did not follow-up with telephone interviews were in relapse.

## Conclusions

This is one of the few studies that investigate the effectiveness of compulsory rehabilitation and voluntary rehabilitation in China. This study revealed that compulsory rehabilitation is as effective as voluntary rehabilitation in (1) getting jobs and increasing monthly income, (2) having a good relationship with family, and (3) becoming more satisfied with their spare time. This study also showed that participant demographic characteristics of participants from CRCs and VRCs were very different and may imply the necessity of these two approaches in China. Employment and family support may prevent relapse. This article provides some evidence that compulsory rehabilitation is still effective and necessary; hence, it should not be abolished.

## Data Availability Statement

The raw data supporting the conclusions of this article will be made available by the authors, without undue reservation.

## Ethics Statement

The studies involving human participants were reviewed and approved by the Ethics Committee of Second Xiangya Hospital of Central South University. Written informed consent to participate in this study was provided by the participants' legal guardian/next of kin.

## Author Contributions

XW conceived and designed the review. KH lead the screening, data extraction, data analysis, and writing of the article. CY provided methodological and substantive support throughout the manuscript process, also as a co-author. ZW, YD, XC, and YH helped with screening, data extraction, and verification. All authors have reviewed and approved the final submission.

## Conflict of Interest

The authors declare that the research was conducted in the absence of any commercial or financial relationships that could be construed as a potential conflict of interest. The handling Editor declared a shared affiliation, though no collaboration, with the authors.

## Publisher's Note

All claims expressed in this article are solely those of the authors and do not necessarily represent those of their affiliated organizations, or those of the publisher, the editors and the reviewers. Any product that may be evaluated in this article, or claim that may be made by its manufacturer, is not guaranteed or endorsed by the publisher.
